# Not all vaginal microbiomes are equal: functional context shapes immune landscapes

**DOI:** 10.1128/mbio.03645-25

**Published:** 2026-02-05

**Authors:** Amanda Williams, Amaury Maros, Michael T. France, Jacques Ravel, Johanna B. Holm

**Affiliations:** 1 Center for Advanced Microbiome Research and Innovation, University of Maryland School of Medicine 12264, Baltimore, Maryland, USA; 2 Institute for Genome Sciences, University of Maryland School of Medicine 189800, Baltimore, Maryland, USA; 3 Department of Microbiology and Immunology, University of Maryland School of Medicine 200790, Baltimore, Maryland, USA; University of California Irvine, Irvine, California, USA

**Keywords:** microbiota, community structure, metagenomic, metatranscriptomics, *Lactobacillus*, vagina, anaerobes, microbial communities, bioinformatics, lactate dehydrogenase, gene expression, immunology

## Abstract

Taxonomic classification alone fails to capture the ecological and functional diversity of vaginal microbiomes, particularly those dominated by *Gardnerella* species. Using the expanded VIRGO2 gene catalog, we developed the vaginal inference of subspecies and typing algorithm (VISTA), a novel ortholog-based framework that defined metagenomic subspecies and 25 metagenomic community state types (mgCSTs), including six distinct *Gardnerella*-dominated profiles. The mgCSTs exhibit marked differences in species composition, functional gene content, transcriptional activity, and host immune responses. These findings reveal that *Gardnerella* predominance does not uniformly equate to dysbiosis and underscore the importance of functional context in shaping host-microbiome interactions. VISTA provides scalable classifiers and an interactive application to support mechanistic studies of vaginal microbiome function and its implications for reproductive health.

**IMPORTANCE:**

The vaginal microbiome plays a central role in reproductive and gynecologic health, yet its functional diversity and ecological organization remain poorly understood. Traditional 16S rRNA approaches provide only a partial view of this complexity, overlooking the strain-level variation that often determines microbial behavior and host outcomes. By applying metagenomic sequencing and scalable computational modeling, we developed the vaginal inference of subspecies and typing algorithm, a framework that defines gene-based subspecies and community state types across diverse populations. These classifications reveal new insights into the genomic and ecological foundations of vaginal community structure and offer a standardized resource for comparative and translational microbiome research. This work establishes the foundation for functionally informed diagnostics and precision interventions targeting women’s reproductive health.

## INTRODUCTION

Unlike other human-associated microbiomes, an optimal vaginal microbiome is characterized by low bacterial species richness and predominance of *Lactobacillus* species (1). In contrast, vaginal microbiomes containing a diverse set of facultative and obligate anaerobic bacteria, such as *Gardnerella*, *Sneathia*, *Prevotella,* and “*Candidatus* Lachnocurva vaginae,” are considered non-optimal and associated with adverse reproductive and urogenital health outcomes (2–7). To describe these patterns of compositional variation, vaginal microbiomes are commonly classified into community state types (CSTs), a taxonomy-based framework defined by the relative abundance of key bacterial taxa. Five primary CSTs that differ in community structure have been described, each defined by its dominant species: *L. crispatus* (CST I), *L. gasseri* (CST II), *L. iners* (CST III), diverse anaerobes (CST IV), and *L. jensenii* (CST V) (8).

Despite its apparent taxonomic simplicity, the vaginal microbiome harbors hundreds of microbial strains that cannot be resolved by marker gene profiling (1, 9–11). Beneath the compositional layer lies a rich functional landscape distributed across multiple strains of the same species, offering deeper ecological and clinical insights into reproductive health. These conspecific genotypic assemblages, termed metagenomic subspecies (mgSs), represent the genomic repertoire of a species within a vaginal microbiome (9, 12). The combination of all mgSs in a given vaginal microbiome defines metagenomic community state types (mgCSTs) (12). Characterizing microbiomes at the mgSs and mgCST level enables a shift beyond taxonomy to functionally defined groupings that more accurately represent host-microbiome functional interactions and are better suited to probing causal relationships. While mgCSTs and VALENCIA CSTs (13) often align when compared using taxonomy alone, mgCSTs represent underlying genomic structuring in the vaginal microbiome that the CST framework does not capture.

With the recent expansion of the VIRGO non-redundant vaginal gene catalog (14), now comprising over 1.7 million genes from more than 280 species, including 15 *Gardnerella* species, updated methods were needed to refine mgSs and mgCST classifications (12). We herein describe the vaginal inference of subspecies and typing algorithm (VISTA), the latest version of the mgCST classifier. A key innovation in VISTA is the use of vaginal orthologous genes (VOGs), clusters of non-redundant genes grouped by protein sequence similarity, to define both mgSs and mgCSTs. Clustering by orthologs rather than individual genes is advantageous because orthologs better reflect the microbiome’s functional capabilities (15), thereby providing a scalable approach to link species- and strain-level diversity to measurable functional outcomes. This approach also enables meaningful cross-species comparisons by grouping genes with shared functions despite sequence variation, effectively simplifying large metagenomic data sets while preserving essential functional information. Furthermore, VISTA mgCSTs proved functionally coherent in downstream analyses: paired metatranscriptomic and host immunological profiling revealed distinct transcriptional activities and immune signatures, underscoring their utility for interpreting microbiome function *in vivo*.

## RESULTS

### Derivation of mgSs and VISTA mgCSTs

**Fig 1:**
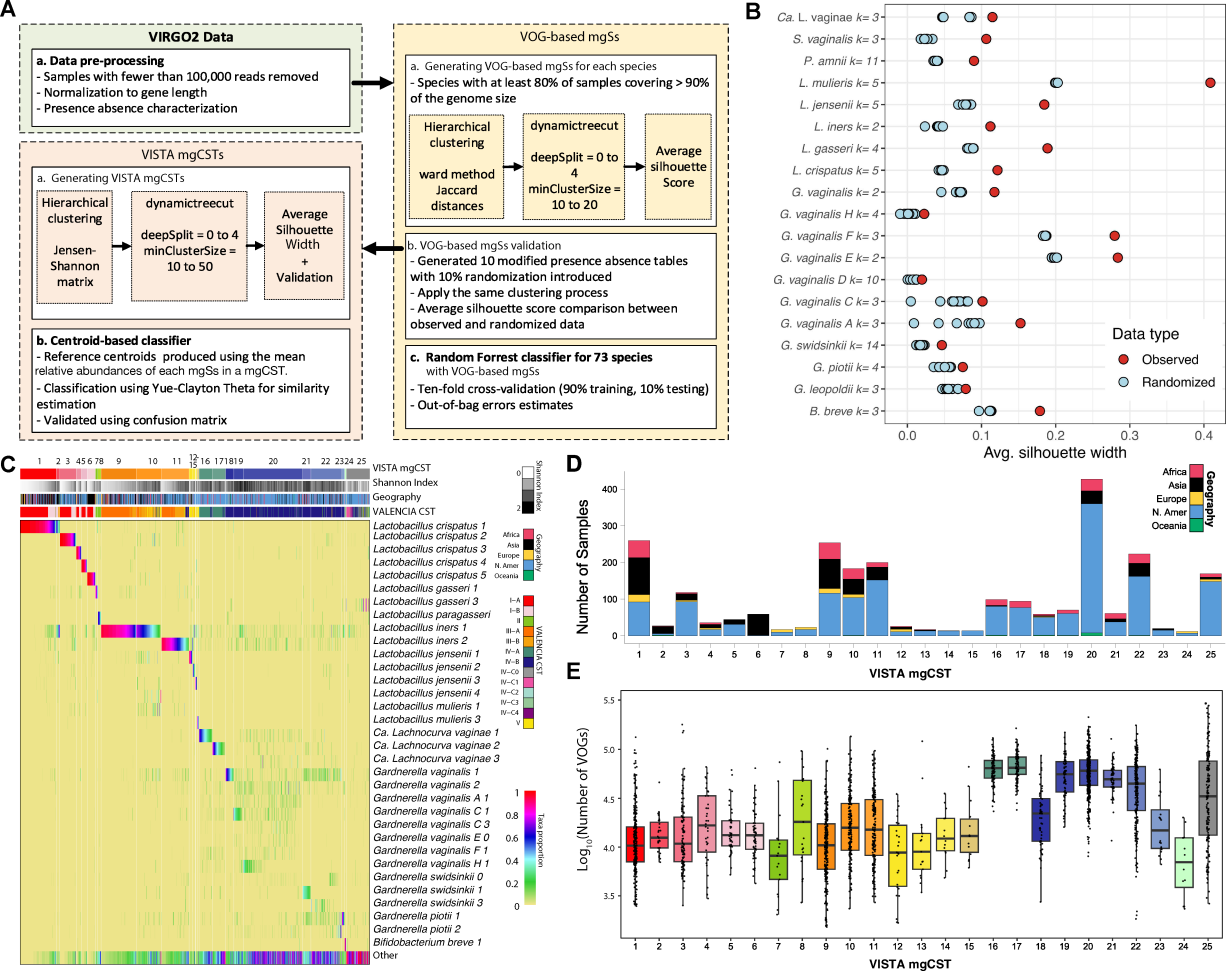
Development, composition, and prevalence of VISTA mgCSTs. (**A**) Flow diagram for construction of VISTA mgCSTs. (**B**) Comparison of VOG-based mgSs clusters generated from the observed and randomized data for selected species. (**C**) MgCST composition and basis of classification. It is important to note that “*Ca.* Lachnocurva vaginae” is referred to as UBA629 sp005465875 in VIRGO2, but as its genome has recently been completed, VISTA uses the newly assigned name. (**D**) Distribution and prevalence of mgCSTs by region where source data have originated. (**E**) The log10-transformed number of VOGs per sample, stratified by mgCST.

VOG-based mgSs and VISTA mgCSTs were derived from the metagenomic data included in VIRGO2 (14) (Fig. 1A). To define mgSs, we focused on species with sufficient genome and sample coverage (present in 10 samples with ≥80% of the genome’s protein-coding genes present in the metagenomic data) and applied hierarchical clustering, selecting optimal clusters through silhouette-based stability testing against randomized data sets (Fig. 1B). Species that did not meet this threshold were still included in mgCST clustering as species-level units, but not as mgSs. This approach was applied to 73 prevalent vaginal species (Fig. S1). VOG content within each species was clustered in a process that implemented dynamic tree cutting to generate the mgSs. To test the stability and significance of the clustering parameters and improve the robustness of the mgSs clustering, 10% randomization was introduced into the data and compared to the original clustering (Fig. 1B; Fig. S1). Across all 73 species, the average silhouette widths of the observed data exceeded those of the randomized data sets, confirming that the derived mgSs represent a non-random genomic structure. The number of mgSs identified per species ranged from 2 to 11, with the largest subdivisions observed for *Prevotella amnii* (k = 11), *Mycoplasma hominis* (k = 10), *Gardnerella vaginalis* D (k = 10), *Prevotella sp000758925* (k = 10), and *Ureaplasma parvum* (k = 10). The largest differences between observed and randomized silhouette widths were evident among all *Lactobacillus* species (*L. crispatus,* k = 5; *L. gasseri,* k = 4; *L. iners,* k = 2; *L. jensenii,* k = 5; *L. mulieris,* k = 5) as well as *Limosilactobacillus* coleohominis (k = 3); *G. vaginalis* subgroups A (k = 3), E (k = 2), F (k = 3), and H (k = 4); *Streptococcus agalactiae* (k = 4); *Varibaculum cambriense* (k = 2); and *Fannyhessea vaginae* (k = 2). In contrast, *Actinomyces christensenii* (k = 7), *Prevotella timonensis* (k = 3), *Mobiluncus indolicus* (k = 7), *Mycoplasma hominis* (k = 10), and *KA00274 sp902373515* (k = 7) exhibited the lowest observed silhouette widths relative to their randomized counterparts.

Samples were then clustered by the relative abundance of species and mgSs to define VISTA mgCSTs, with parameters optimized using silhouette width to balance resolution and stability (Fig. S2). In total, 25 mgCSTs were identified (Fig. 1C), including those predominated by *Lactobacillus*: (*L. crispatus*, mgCSTs 1–6; *L. gasseri*, 7; *L. paragasseri*, 8; *L. iners*, 9–11; *L. jensenii*, 12–14; *L. mulieris*, 15), as well as “*Ca*. L. vaginae” (16 and 17, GTDB Taxonomy: UBA629), *G. vaginalis* (18), *G. vaginalis* C (19), *G. vaginalis* H (20), *G. swidsinkii* (21 and 22), *G. piotii* (23), *Bifidobacterium breve* (24), and others (25).

### VISTA mgCST geographic distribution

The geographic distribution of VISTA mgCSTs revealed pronounced regional patterns across five continents (Fig. 1D). MgCST 1 (*L. crispatus*) was broadly distributed across Asia (*n* = 101), North America (*n* = 92), and Africa (*n* = 47), whereas mgCST 6 (*L. crispatus*) was almost entirely restricted to Asia, predominantly Bangladesh. *L. iners* mgCSTs 10–11 were common in North America and Asia, while mgCST 8 (*L. paragasseri*) was largely North American. Among *Gardnerella*-dominated communities, mgCST 21 (*G. swidsinskii*) was highly prevalent in North America (*n* = 353) but also present in Africa and Asia, whereas mgCST 20 (*G. vaginalis* H) and mgCST 19 (*G. vaginalis* C) were primarily North American. “*Ca*. L. vaginae” mgCSTs 16–17 were almost exclusively North American, while less common mgCSTs, including *B. breve* (24) and “Other” (25), were sparsely distributed.

### Functional (VOG) richness across VISTA mgCSTs

Functional richness, measured as the number of VOGs per sample, varied considerably among VISTA mgCSTs (Fig. 1E). MgCSTs 16 and 17 (“*Ca*. L. vaginae”) contained the most VOGs (~6.53e4 and 7.05e4, respectively; SD ≈ 2.14e4), reflecting consistently broad functional repertoires. Among *Lactobacillus*-dominated mgCSTs, mgCST 4 (*L. crispatus*; ~2.18e4; SD ≈ 1.68e4), mgCST 8 (*L. paragasseri*; ~2.85e4; SD ≈ 2.64e4), and mgCSTs 9–11 (*L. iners*; ~2.20e4; SD ≈ 1.90e4) displayed moderately high functional content. Within *Gardnerella*-dominated mgCSTs, mgCSTs 18 (*G. vaginalis*; ~2.46e4; SD ≈ 1.74e4) and 23 (*G. piotii*; ~2.05e4; SD ≈ 1.52e4) exhibited the lowest richness compared to other *Gardnerella* communities. MgCSTs 19 (*G. vaginalis* C; ~5.81e4; SD ≈ 2.77e4) and 20 (*G. vaginalis* H; ~6.35e4; SD ≈ 2.89e4) contained the most VOGs, with mgCST 20 showing particularly high variability. MgCSTs 21 and 22 (*G. swidsinskii*; ~5.23e4; SD ≈ 1.81e4 and ~4.84e4; SD ≈ 3.11e4, respectively) had substantial functional content, with mgCST 22 being the most variable. MgCST 24 (*B. breve*) had the lowest richness (~8.60e3; SD ≈ 6.10e3), while the heterogeneous “Other” mgCST 25 exhibited elevated yet highly variable (~5.17e4; SD ≈ 5.51e4).

### Classifier performance and implementation

To enable robust and scalable assignment of metagenomic samples to mgSs and VISTA mgCSTs, we developed and validated machine learning classifiers using VOG-based genomic signatures. For mgSs, random forest models were trained to predict subspecies identity from VOG composition. Performance was assessed using out-of-bag (OOB) error rates, which were low across most taxa—86% of mgSs exhibited OOB error <0.2 (Fig. S3A), demonstrating that VOG-based features provide strong discriminatory power for subspecies-level classification. Notably, *Lactobacillus* and *Gardnerella* species were predicted with particularly high accuracy: *L. crispatus*, *L. iners*, and all *Gardnerella* genomospecies, except *G. vaginalis* D, showed OOB error <0.2, confirming that VOG-informed clustering captures biologically meaningful intraspecies structure. In contrast, the classification performance for certain *Prevotella* species was more variable, consistent with the elevated genomic heterogeneity within this genus.

For higher-level community classification, centroid-based reference models were used to assign mgCSTs, achieving strong concordance with hierarchical clustering results (κ = 0.90, 95% CI: 0.89–0.91). The corresponding confusion matrix exhibited a dense diagonal (Fig. S3B), reflecting close agreement between predicted and observed mgCSTs. Misclassifications were rare and typically occurred among closely related or functionally similar communities (e.g., mgCSTs 10 and 11), suggesting that the classifier maintained biologically relevant continuity between adjacent community types. Together, these results confirm that the VOG-based framework implemented in VISTA yields accurate, generalizable, and biologically coherent mgSs and mgCST assignments, supporting its application to new vaginal metagenomic data sets.

### VISTA mgCST diversity and composition

Though VISTA mgCSTs and VALENCIA CSTs are largely congruent using taxonomy alone (Fig. S4), VISTA mgCSTs revealed genetic diversity of the vaginal microbiome not captured by the CST framework. For example, within VALENCIA CST III, which is broadly characterized by *L. iners* dominance, VISTA identified three discrete mgCSTs (9–11) distinguished by the underlying *L. iners* mgSs genetic composition and the co-occurrence of secondary taxa representing functionally divergent ecological states within CST III. Similarly, microbiota assigned to VALENCIA CST IV, which encompasses diverse, non-*Lactobacillus*–dominated communities, was partitioned into multiple “*Ca*. L. vaginae” (16–17) and *Gardnerella*–enriched (18–23) mgCSTs, each exhibiting distinct genetic contexts and functional capacities.

A total of 3,935 VOGs ascribed to *L. iners* produced two mgSs dominating mgCSTs 9–10 (mgSs 1) or mgCST 11 (mgSs 2). Those with mgSs 2 contained more VOGs on average than mgSs 1 (1,411 versus 1,290, respectively). A set of 82 gene orthologs present in mgSs 2 but absent from mgSs 1 included plasmid-associated conjugation and maintenance proteins (TrbC, DNA-entry nuclease; ParR, replication initiation protein), mobile element and recombination-associated proteins (IS607/IS200/IS605 family transposases, Rpn family recombinases, and XerS tyrosine recombinases), DNA modification and restriction enzymes (DNA methylase N-4/N-6 domain-containing protein, class I SAM-dependent methyltransferase, and restriction endonuclease subunit M), surface-anchored or secretion system proteins (LPXTG-motif proteins, VaFE repeat-containing proteins, type IV and type VII secretion system components, Class A sortase, signal peptidase I, and SecA/SecY protein translocases), and chromosomal genes involved in core cellular processes (penicillin-binding protein, single-stranded DNA-binding protein, helix–turn–helix DNA-binding proteins, HU family DNA-binding protein, integral membrane proteins, S1 motif-containing proteins, M23 family metallopeptidase, DUF87 domain-containing proteins, and VWFA domain-containing proteins; Table S1).

**Fig 2:**
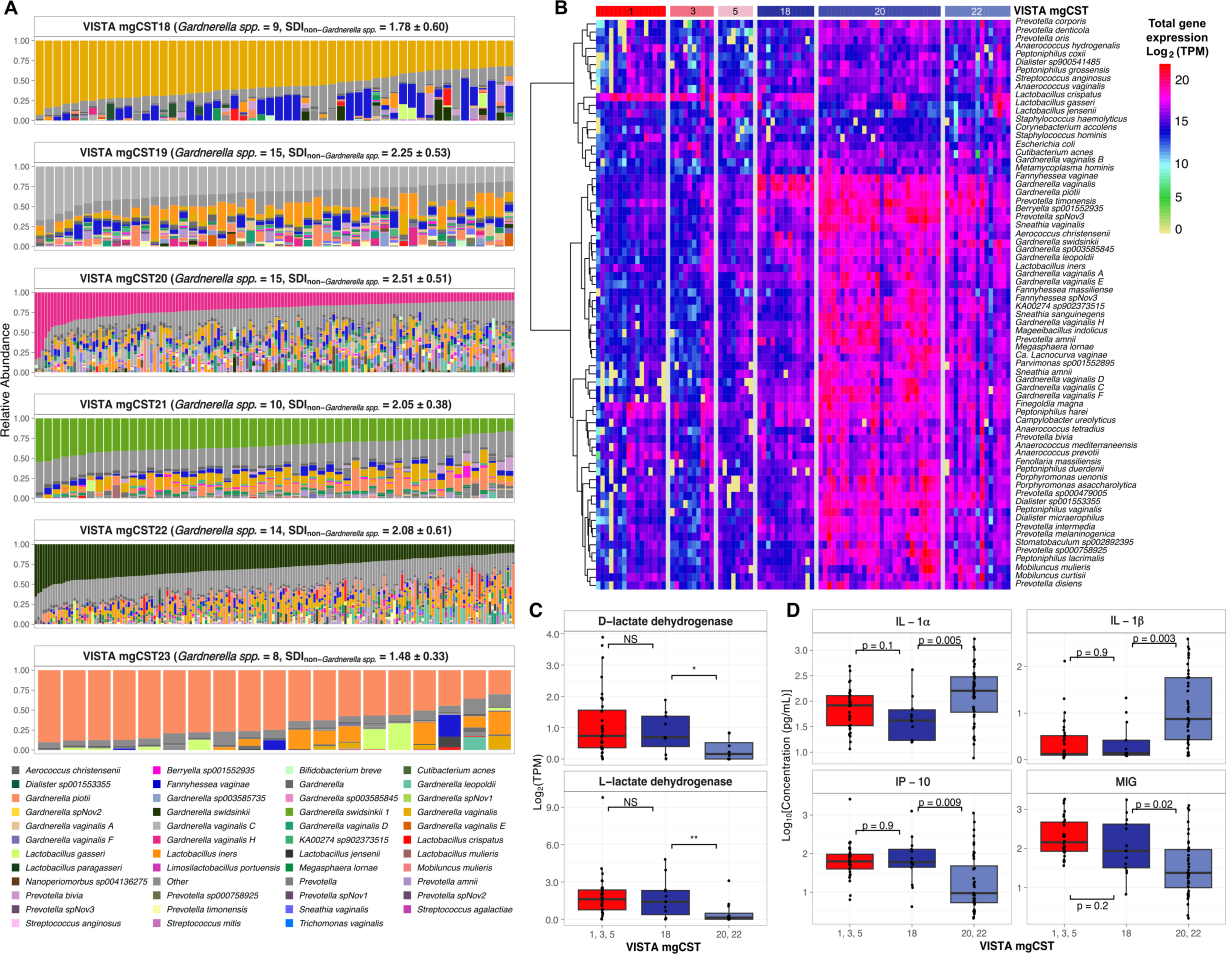
Distinctive features of *Gardnerella*-predominated vaginal microbiomes across VISTA mgCSTs. (**A**) Proportion of samples with each *Gardnerella* VOG cluster in VISTA mgCSTs dominated by *Gardnerella* species. At least half of the VOGs comprising a VOG cluster had to be present in a sample to be included. The row labels are the species of *Gardnerella* and the VOG cluster number. (**B**) Heatmap displaying the 70 most metabolically active microbial species across samples with available metatranscriptomic data from *Lactobacillus crispatus*- or *Gardnerella*-dominated mgCSTs. Samples are grouped by mgCST, and only mgCSTs with ≥5 samples were included in the analysis. Gene expression was transcripts per million (TPM)-normalized, summed across all genes within each species, log2-transformed, and hierarchically clustered using Ward’s method. (**C**) Expression of *L. crispatus* D- and L-lactate dehydrogenase genes (D-LDH: VOG0191398; L-LDH: VOG0188438, VOG0186335, and VOG0189631) across mgCSTs. (**D**) Log10-transformed concentrations (pg/mL) of immune markers interleukin-1α (IL-1α), interleukin-1β (IL-1β), interferon gamma-induced protein 10 (IP-10), and monokine-induced gamma interferon (MIG) in the same samples profiled using metatranscriptomics, grouped by mgCST. ANOVA-derived *P*-values indicate comparisons between mgCST 18 and *L. crispatus*-dominated mgCSTs (1, 3, and 5) and between mgCST 18 and *Gardnerella*-dominated mgCSTs (20 and 22) for each immune marker.

*Gardnerella*-enriched VISTA mgCSTs (18–23) exhibited marked heterogeneity in species composition, ecological structure, and genomic potential (Fig. 2A). MgCST 18 and 23 were the least taxonomically diverse, dominated almost exclusively by *G. vaginalis* mgSs 1 and *G. piotii*, respectively. In contrast, mgCSTs 19–22 harbored multiple *Gardnerella* species alongside diverse anaerobes such as *Prevotella*, *Fannyhessea*, *Dialister*, and *Megasphaera*, reflecting increased species richness and evenness. Shannon diversity indices (SDI) of non-*Gardnerella* taxa were lowest in mgCST 18 (SDI = 1.78 ± 0.60) and 23 (SDI = 1.48 ± 0.33) and highest in mgCST 19 (SDI = 2.51 ± 0.63) and 20 (SDI = 2.51 ± 0.61), consistent with pronounced ecological stratification across these communities. MgCSTs 21 and 22, both dominated by *G. swidsinkii*, differed in mgSs composition—mgCST 21 microbiomes contained only *G. swidsinkii* mgSs 1, whereas those in mgCST 22 did not contain mgSs 1 and instead contained other *G. swidsinskii* mgSs, most frequently mgSs 3, indicating intraspecies diversification. Collectively, these patterns reveal that *Gardnerella*-associated mgCSTs encompass a continuum of community complexity, ranging from near-monospecies dominance to polymicrobial assemblages with extensive strain heterogeneity.

To delineate mgSs differentiation among *Gardnerella* species, we identified gene orthologs that were present in ≥50% of metagenomes within one mgSs but absent in >75% of metagenomes in the other. In *G. swidsinskii*, this comparison highlighted a set of genes unique to mgSs 3 that were enriched for mobile- and phage-associated functions, including helix–turn–helix transcriptional regulators, ParA chromosome partitioning proteins, plasmid maintenance and antidote systems, TolA-like colicin import machinery, and a phage-like integrase (Table S2). For *G. vaginalis*, mgSs 1 encoded a richer complement of restriction–modification and CRISPR-associated systems (Cas1, Cas2, and type I/III endonucleases), along with sugar-binding and transport proteins, α-amylases, two additional pullulanases, and energy metabolism enzymes such as succinate dehydrogenase (Table S3). In contrast, mgSs 2 was enriched for toxin–antitoxin systems, ABC transporters, multidrug resistance loci, surface-anchored adhesins, and conjugative or integrase-associated genes. *G. vaginalis* mgSs 1 was detected primarily within *L. crispatus*-dominated communities mgCSTs, whereas mgSs 2 occurred more frequently in non-optimal, polymicrobial mgCSTs (Table S4).

### Metatranscriptomic activity and functional expression patterns in VISTA mgCSTs

For a subset of samples used to define VISTA mgCSTs, matched metatranscriptomic data enabled analysis of community-level transcriptional activity and species-specific expression (Fig. S5; Fig. 2B and C). Total microbial transcriptional output, calculated as the log10-transformed sum of TPM-normalized gene expression per sample, varied markedly across mgCSTs (Fig. S5). The overall activity was lowest in *L. crispatus*–dominated mgCSTs 1, 3, and 5 (mean = 8.44 ± 0.47; *n* = 34) and highest in the polymicrobial *Gardnerella* mgCSTs 20 and 22 (mean = 9.27 ± 0.41; *n* = 43). The *G. vaginalis*–dominated mgCST 18 showed intermediate activity (mean = 8.84 ± 0.41; *n* = 13), higher than that of *Lactobacillus* communities but lower than that of the more transcriptionally rich, polymicrobial *Gardnerella* groups. No matched data were available for mgCST 23.

The distribution of species-level activity was also evaluated with these samples (Fig. 2B). Among the 70 most transcriptionally active taxa shown in the heatmap, mgCST 18’s expression was tightly concentrated: *G. vaginalis*, *G. piotii*, and *Fannyhessea vaginae* accounted for the bulk of transcript abundance in these samples. Quantitatively, these three species together contributed ~29.93% ± 9.96% of the total transcriptional pool in mgCST 18 samples (*G. vaginalis* ~15.55% ± 5.99%, *G. piotii* ~2.90% ± 2.23%, and *F. vaginae* ~10.02% ± 9.11%). In contrast, *L. crispatus*–dominated mgCSTs showed ~27.19% ± 16.07% of all transcripts attributable to *L. crispatus*. In mgCSTs 20 and 22, transcription was more evenly partitioned. The dominating *Gardnerella* lineages accounted for 2.36% ± 3.67% (mgCST 20, *G. vaginalis* H) and 2.56% ± 2.15% (mgCST 22, *G. swidsinkii*) of the total expression. However, total *Gardnerella* transcription reached 17.08% ± 13.79% in mgCST 20 and 14.72% ± 9.14% in mgCST 22, indicating that the overall *Gardnerella* activity remained high despite the relatively modest contributions from the dominating species. In these mgCSTs, co-occurring anaerobes in the genera *Prevotella*, *Fannyhessea*, and *Sneathia* displayed elevated expressions, accounting for much of the transcriptional activity (mgCST 20: 16.31% ± 5.82%, 3.20% ± 2.58%, and 3.57% ± 2.78%; mgCST 22: 13.43% ± 8.50%, 1.84% ± 1.49%, and 1.70% ± 1.44%, respectively).

Several low-abundance taxa, notably *Finegoldia magna* and *Peptoniphilus harei*, contributed disproportionately to transcript pools across mgCSTs. In mgCSTs 1, 3, and 5, *F. magna* accounted for 0.3%–9.3% and *P. harei* for 0.1%–5.6% of total microbial transcription. In mgCST 18, these species contributed 0.1%–4.1% and 0.1%–1.7% of total transcription, respectively, while in mgCSTs 20 and 22, contributions ranged from 0.1% to 4.9% for *F. magna* and 0.1% to 3.2% for *P. harei*.

Although present at low relative abundance in several *Gardnerella*-dominated communities, *L. crispatus* remained transcriptionally active in mgCST 18, accounting for 3.59% ± 2.22% of the total transcriptional activity. Within this transcriptional output, both the D- and L-lactate dehydrogenase genes (D-LDH: VOG0191398; L-LDH: VOG0188438, VOG0186335, and VOG0189631) were significantly upregulated in mgCSTs 1, 3, and 5 relative to mgCSTs 20 and 22 (D-LDH log2FC = 2.87, *P* = 0.004; L-LDH log2FC = 5.62, *P* = 0.03; Table S5). The *L. crispatus* mgSs present in mgCST 18 comprised a mixture of mgSs 1 (typically dominant in mgCST 1) and 0, whereas only mgSs 0 was observed in the other *Gardnerella*-dominated mgCSTs (Table S6).

### Host immunological states and VISTA mgCSTs

We compared the concentrations of four immune mediators (IL-1α, IL-1β, IP-10, and MIG) across VISTA mgCSTs dominated by *Lactobacillus crispatus* (mgCSTs 1, 3, and 5), *Gardnerella vaginalis* (mgCST 18), and other *Gardnerella*-dominated mgCSTs 20 and 22 (Fig. 2D; Fig. S6). A multivariate analysis of variance (MANOVA) showed no significant difference in the overall immune profiles between *L. crispatus*–dominated mgCSTs and mgCST 18 (Pillai’s trace = 0.0998, F_4,42_ = 1.16, *P* = 0.34). However, a significant multivariate effect was observed between mgCST 18 and the other *Gardnerella*-dominated mgCSTs (Pillai’s trace = 0.3125, F_4,52_ = 5.91, *P* = 0.00054). Univariate ANOVAs indicated that mgCST 18 had significantly lower concentrations of all four cytokines—IL-1α (F = 8.74, *P* = 0.0046), IL-1β (F = 9.89, *P* = 0.0027), IP-10 (F = 6.91, *P* = 0.011), and MIG (F = 5.47, *P* = 0.023)—compared to the other *Gardnerella* mgCSTs. None differed significantly between mgCST 18 and the *L. crispatus* mgCSTs (all *P* > 0.13).

## DISCUSSION

The vaginal microbiome is integral to reproductive health, yet its structure and function remain poorly resolved. While 16S rRNA surveys have advanced taxonomic profiling, they cannot capture the strain-level or functional variation that drives ecological and clinical outcomes. Metagenomic sequencing enables this higher-resolution view, linking gene content to community structure and host interactions. However, standardized frameworks for functional, scalable microbiome classification are lacking. To address this, we developed VISTA, a metagenomic tool that defines VOG-based subspecies (mgSs) and community state types (mgCSTs), yielding 25 distinct mgCSTs across global populations.

We observed an imbalance in the country of origin for some VISTA mgCSTs, including mgCST 6 (predominated by *L. crispatus* mgSs 5), wherein 97% of samples were from Bangladesh. This pronounced regional confinement suggests that host genetics, environmental factors, or population-specific selective pressures may influence the persistence or dominance of certain *L. crispatus* lineages. However, such geographic restrictions underscore the need for regionally representative reference databases. While VIRGO2 and VISTA represent a substantial expansion over earlier frameworks, the current reference set remains limited in life stage (excluding pre-pubescent and post-menopausal individuals), race and ethnicity, pregnancy status, and sexual or infectious health histories, highlighting the need to incorporate more globally and demographically diverse populations to capture the full spectrum of vaginal microbiome variation (16, 17).

Functional capacities vary substantially both within and between VISTA mgCSTs. *Lactobacillus* mgSs (mgCSTs 1–15) generally encoded fewer VOGs, likely reflecting lower metagenomic subspecies richness and/or greater functional redundancy. In contrast, mgCSTs 16–25 exhibit broader functional repertoires, consistent with increased microbial diversity and ecological complexity. MgSs-level differences illustrate how sub-species genetic variation shapes both function and ecology. The two *L. iners* mgSs detected across mgCSTs 9–11 showed clear subspecies-level divergence. MgSs 2 (mgCST 11) contained a broader suite of plasmid-associated genes, mobile elements, and chromosomal loci than mgSs 1, indicating greater genomic plasticity and a higher potential for horizontal gene transfer. MgSs 2 was also found enriched in surface-anchored proteins, adhesion factors, and secretion system components, potentially facilitating interactions with host tissues or neighboring microbes, which may enhance survival in more diverse communities like CST IV. These additions to both its plasmid and chromosomal gene pools suggest that *L. iners* populations within CST III are not functionally uniform but differ in the genome architecture and interaction potential, which may help explain the ecological distinctions between mgSs 1- and mgSs 2-dominated communities.

The wide range in VOG counts across *Gardnerella-*predominated VISTA mgCSTs (18–23) reflects underlying differences in species composition and strain-specific ortholog content, suggesting that *Gardnerella* strains contribute unevenly to the functional capacity of vaginal microbiomes. These differences are further shaped by sample-level variation in the number of *Gardnerella* species and the richness and evenness of co-occurring taxa. For example, mgCSTs 19 and 20 contained the most *Gardnerella* species and the greatest SDI of non-*Gardnerella* species, indicating particularly complex microbial environments shaped by niche partitioning and/or functional differentiation (12).

Although VISTA mgCSTs 21 and 22 were both dominated by *G. swidsinskii*, their strain-level composition differed markedly, with mgCST 21 containing only *G. swidsinskii* mgSs 1, whereas mgCST 22 microbiomes included many mgSs, especially mgSs 3. The enrichment of genes linked to mobile genetic elements suggests heightened genomic plasticity, enabling horizontal gene transfer and acquisition of adaptive traits (16). Such capabilities likely confer ecological advantages, influencing strain persistence, competitive interactions, and potential pathogenicity within the host environment (17). These functional capacities may enhance the ecological competitiveness of *G. swidsinskii* mgSs 3, possibly shaping its persistence and interactions within the host environment.

VISTA mgCSTs 18 and 23 emerged as the least taxonomically diverse *Gardnerella-*predominated mgCSTs, characterized by a pronounced dominance of either *G. vaginalis* mgSs 1 or *G. piotii*. This was reflected in their reduced functional diversity, as evidenced by significantly fewer VOGs per sample compared to the more taxonomically complex mgCSTs 19–22. For samples classified as mgCST 18 with matched metatranscriptomic data, total microbial gene expression (summed expression of all genes from all species present) was lower in mgCST 18 than in mgCST 20, and comparable to, but less varied than, mgCST 22. Within mgCST 18, *G. vaginalis*, *G. piotii*, and *Fannyhessea vaginae* were the most transcriptionally active species. By contrast, the more diverse mgCSTs 20 and 22 exhibited elevated expression across a broader range of taxa, including all identified *Gardnerella* species as well as species of *Prevotella*, *Dialister*, *Porphyromonas*, *Megasphaera*, and *Berryella sp001552935*, consistent with more complex metabolic interactions.

We hypothesized that these transcriptional profiles might reflect distinct immunological landscapes. Non-optimal communities are typically associated with reduced IP-10 and MIG, as well as elevated concentrations of IL-1α and IL-1β, compared to optimal, *Lactobacillus-*predominated communities (18, 19). Indeed, significant differences in these immune markers were observed between VISTA mgCST 18 and other *Gardnerella* mgCSTs. Interestingly, mgCST 18 displayed an immune profile more similar to that of *L. crispatus*-dominated mgCSTs 1–6 than to the other *Gardnerella*-dominated mgCSTs. This finding suggests that, despite its *G. vaginalis* predominance, VISTA mgCST 18 may share immunological features with optimal states. These associations were corroborated by immune marker data from an independent study incorporated into VIRGO2 (PRJNA797778), which recapitulated the same trends and reinforced the distinct immunological profile of mgCST 18.

The predominance of *G. vaginalis* in VISTA mgCST 18, coupled with its narrower transcriptional activity across taxa, suggests a competitive ecological dynamic characterized by niche monopolization (20). In contrast, the broader transcriptional profiles and higher diversity indices observed in mgCSTs 20 and 22 are consistent with niche partitioning, wherein multiple taxa contribute to community function through differentiated metabolic roles. These ecological distinctions may underlie the immunological differences observed across *Gardnerella*-predominated VISTA mgCSTs. The distinctiveness of mgCST 18 may be explained by the genetic repertoire of *G. vaginalis* mgSs 1, which suggests a strategy of genomic stability, host specialization, and enriched carbohydrate metabolism mechanisms supporting survival in a low-pH, resource-limited milieu. Although pullulanase is broadly conserved across *Gardnerella* species, the presence of two additional pullulanase genes in mgSs 1 suggests an expanded capacity for glycogen utilization as changes in pullulanase architecture could affect nutrient access (21). Notably, mgSs 1 also encoded an L-lactate permease, enabling uptake of exogenous L-lactate (e.g., produced by *Lactobacillus*) and potentially facilitating coexistence with *L. crispatus*. Equally distinctive is mgSs 1’s genomic defense architecture. A Type I restriction–modification system (specificity subunit S, with adenine- and cytosine-specific DNA methyltransferases) operates alongside a Type I-E CRISPR–Cas module (Cas1, Cas2, and CasE). Together, these systems create a strong barrier to horizontal gene flow, preserving genome structure integrity (22). Such genomic insulation may underlie exclusionary dominance and stability of mgCST 18. In contrast, *G. vaginalis* mgSs 2 encoded a broader array of transporters, peptidases, and mobile elements, consistent with metabolic opportunism and polymicrobial interactions. These differences suggest that mgSs 2 may exhibit greater stress resilience and host interaction potential, whereas mgSs 1 retains more canonical metabolic and defense machinery, reflecting divergent ecological strategies within *G. vaginalis*. Accordingly, *G. vaginalis* mgSs 1 was uniquely observed in optimal mgCSTs (e.g., *L. crispatus*-dominated), whereas *G. vaginalis* mgSs 2 predominated in non-optimal, polymicrobial communities where metabolic flexibility and interspecies interactions are advantageous.

Intriguingly, despite the low abundance of *Lactobacillus* in mgCST 18, *L. crispatus* exhibited elevated gene expression compared to other *Gardnerella*-dominated mgCSTs. Expression of D- and L-lactate dehydrogenase was comparable to that observed in *L. crispatus*-dominated mgCSTs and much higher than in mgCSTs 20 and 22, aligning with the lactate permease–mediated compatibility inferred for *G. vaginalis* mgSs 1. This observation is supported by the immune marker profiles, wherein mgCST 18 showed signals distinct from other *Gardnerella*-dominated mgCSTs, with higher IP-10 and MIG, markers positively correlated with vaginal *Lactobacillus* abundance (18, 19, 23), and lower IL-1α and IL-1β, cytokines associated with adverse reproductive health outcomes and the depletion of *D*-lactic acid-producing *Lactobacillus* species (18, 24). These findings suggest that mgCST 18 may interact with host immunity more similarly to *L. crispatus-*dominated states than to other *Gardnerella* communities. More broadly, across all mgCSTs, including those dominated by *Lactobacillus*, low-abundance species maintained disproportionately high transcriptional activity, underscoring their functional importance irrespective of their relative abundances (25). In particular, *Finegoldia magna* and *Peptoniphilus harei* were metabolically active across all mgCSTs. Collectively, mgCSTs provide an ecological and functional framework for understanding the metabolic potential and stability of distinct vaginal microbial communities, shedding light on the dynamic interactions that shape vaginal health and dysbiosis.

VISTA was trained on high-quality metagenomic data to enable robust assignment of mgSs using random forest classifiers and mgCSTs using centroid-based reference models, achieving strong concordance with hierarchical clustering. To support integration into downstream analyses, the mgSs and mgCST classifiers are implemented within a single R script that accepts VIRGO2-derived outputs (see Fig. S3C). Although VISTA does not require assembled genomes, accurate classification depends on sufficient sequencing depth in the input metagenomic data to robustly detect and quantify VIRGO2 genes, and samples with sparse coverage may yield less confident mgSs or mgCST assignments. Because VISTA reports Yue–Clayton θ for each assignment, users can evaluate classification confidence directly as θ values decrease when low sequencing depth compromises estimation of mgSs relative abundances. Additional guidance on interpreting θ scores, as well as recommendations regarding the minimum number of VIRGO2-mapped genes required for reliable mgCST assignment, is provided in the VISTA GitHub documentation.

To enhance interpretability and facilitate exploratory data analysis, we also developed a Streamlit-based application with interactive modules. One module visualizes VOG-based mgSs using coverage plots and presence/absence heatmaps; another enables the exploration of mgCSTs, including sample distributions and comparisons to the original mgCST framework; and a third summarizes VOG cluster prevalence across data sets. The application provides a user-friendly interface for running the mgCST classifier powered by Plotly for interactive visualizations. The code for the tool is available at https://github.com/JHolm-Lab/VISTA.

In conclusion, VISTA’s VOG-based clustering approach represents a significant advance in vaginal microbiome research and demonstrates that even within a single species, different mgSs can carry distinct functional repertoires. By focusing on functional orthologs rather than individual genes, VISTA provides improved subspecies resolution and enables more precise exploration of functional associations between vaginal microbiome composition and health outcomes. While continued refinement of VOG-based mgSs and mgCST classifications will be important for species with complex and evolving pangenomes, VISTA’s robust performance across diverse microbial species, its adaptability to geographic variation, and the accessibility of its web application establish it as a powerful tool for advancing reproductive health research.

## MATERIALS AND METHODS

### Data processing

Samples with fewer than 100,000 reads mapped to VIRGO2 were excluded from the analysis (*n* = 41), resulting in a final data set comprising 1,625,921 genes (rows) and 2,528 samples (columns). From VIRGO2 annotation files (geneLength and Taxa), gene coverage was calculated by multiplying the number of reads by 150 and dividing by the gene length. A threshold of 0.5 was used to determine the presence or absence of a gene in each sample and create a presence-absence table for each species. Using the VIRGO2 vaginal orthologous gene annotation file, a presence-absence table of VOGs was constructed, with at least one gene present to consider the presence of a VOG.

### VOG-based mgSs

MgSs candidates were defined as those with abundances detected at the species level and with at least 80% of samples covering 90% or more of the genome size according to the Genome Taxonomy Database. Using the presence-absence of VOGs for each candidate, mgSs were generated through hierarchical clustering (Ward method) of Jaccard distances on binary counts, using the vegdist function from the vegan package (version 2.6-8) in R (version 4.4.0). Initial mgSs clusters were defined using the dynamicTreeCut algorithm (version 1.63.1) and the optimal combinations of minClusterSize and deepSplit parameters (varying from 10 to 20 and from 0 to 4, respectively) regarding the average silhouette width determined by the cluster.stats function from the cluster package (version 2.1.6). To validate the clustering approach, we generated 10 additional presence-absence tables per species, each with a random modification of 10% of VOG presence-absence data. Taxa with observed average silhouette scores lower than those generated by randomized tables were re-clustered, reducing the number of mgSs by one at a time until the observed silhouette score exceeded any of the silhouette scores from the randomized tables for that taxon. This applied to six species: *Cutibacterium acnes*, *Fannyhessea vaginae*, *Finegoldia magna*, *Gardnerella vaginalis A*, *KA00274 sp902373515*, and *Prevotella timonensis*. MgSs were visually evaluated for low species coverage. For each species, heatmaps of VOG presence-absence were constructed.

### VOG-based VISTA mgCSTs

VISTA mgCSTs were defined by hierarchical clustering (Ward method) of all samples by mgSs and species proportions. The number of mgCSTs was defined using the dynamictreecut algorithm (version 1.63.1) with deepSplit of 4 (highly sensitive to small clusters), and the optimal minClusterSize (varying from 10 to 20) was determined based on maximizing the average silhouette width.

### Random forests for mgSs classification

Random forests were constructed for each of the 73 species using VOG presence-absence tables as training sets, following the procedure of Holm et al. (12). The randomForestSRC package (version 3.3.1) in R was employed for this analysis. Fine-tuning of parameters was conducted for key taxa, including *Gardnerella vaginalis, Gardnerella swidsinkii, Gardnerella piotii, Lactobacillus crispatus, Lactobacillus gasseri, Lactobacillus iners, and Lactobacillus jensenii*. A 10-fold cross-validation was performed for each taxon, using a 90% training and 10% test split. The misclassification errors of the mgSs random forest model were calculated.

### VISTA

Classification of mgSs and mgCSTs via VISTA uses the approach previously described (12), including hierarchical clustering, and reference centroids derived from the mean relative abundances of each mgSs within a mgCST. Of note, when VOGs from a mgSs are detected in a metagenomic sample but do not have ≥80% of the genome’s protein-coding genes, the species is retained and labeled as mgSs 0, ensuring that low-abundance or partially observed taxa remained represented without overstating the subspecies resolution. For mgCST classification, sample similarity to reference centroids was calculated using Yue-Clayton’s θ, which emphasizes high-abundance mgSs and provides a confidence measure for assignment. Samples were assigned to the mgCST with the highest similarity, and ten-fold cross-validation was performed by generating centroids from each training set and assigning test samples accordingly. A heatmap of mgCSTs (rows as mgSs and columns as samples) and a confusion matrix from cross-validation were produced. Misclassification error was calculated as the average proportion of incorrect assignments across folds, and weighted kappa statistics were used to estimate the concordance between mgCSTs assigned by hierarchical clustering (expected) and nearest-centroid classification (observed).

VISTA requires two arguments to run: (i) the direct output from VIRGO2 after the mapping and compilation steps; (ii) the path to the VISTA master directory. Given the large data volumes generated by VIRGO2, VISTA employs parallel computation to prevent memory saturation. When run on a MacBook M3 Pro using a VIRGO2 output of 2.9 GB containing 1,255,390 genes and 580 samples, the classifier achieved a mean runtime of 17 min.

### Metatranscriptomic sequencing

The methods for library sample collection, RNA extraction, library prep, and sequencing are described in France et al. (25) Reads were mapped to VIRGO2. Low-abundance genes (fewer than 30 reads across all samples) were removed, and the remaining transcripts were normalized using the TPM method. The genes within each species were summed to represent that species, and species that lacked counts in 90% of samples or were absent from the metagenomic sequencing data were removed. The 70 species with the highest transcript expression levels were log2-transformed and plotted. A mgCST required at least 5 samples to be plotted. For comparisons of specific genes like LDH, gene sequences were verified using the canonical sequences from UniProt and KEGG via BLAST prior to normalization. Gene expression values were then normalized to TPM and then adjusted relative to the single-copy *recA* housekeeping gene (VOG0190358). Differential expression of these genes was subsequently assessed using DESeq2 (version 1.42.1). See Table S7 for metatranscriptomic data and corresponding mgCSTs.

### Immune marker measurements

Immunological marker concentrations were measured from vaginal swab eluates using a multiplex bead-based Luminex assay (Luminex Corp, Austin, TX). Concentrations of IL-1α, IL-1β, IP-10, and MIG were quantified simultaneously using fluorescently labeled microspheres conjugated to specific capture antibodies, enabling high-throughput detection from small sample volumes. A mgCST had to have at least 5 samples to be included in the analysis. The concentrations of these immune markers were log10-transformed. See Table S7 for immune marker data and corresponding metatranscriptomic data and mgCSTs.

### Statistical analysis

The SDI was computed using the vegan package (version 2.6-8) in R, with the natural logarithm (log base *e*) as the default. To determine the similarity between mgCST 18, the *L. crispatus* mgCSTs, and the remaining *Gardnerella*-dominated mgCSTs for the immune marker profiles, MANOVA tests were applied to each comparison (mgCST 18 vs *L. crispatus* mgCSTs and mgCST 18 vs *Gardnerella*-dominated mgCSTs). The Pillai test was then applied to evaluate the multivariate significance of the differences between each comparison. ANOVA tests were applied to each individual immune marker to compare mgCST 18 to the *L. crispatus* mgCSTs and *Gardnerella*-dominated mgCSTs.

## Data Availability

All metagenomic data used in VIRGO2 (14) are publicly available from either the NCBI SRA or from the European Nucleotide Archive using the accession numbers provided in here: https://static-content.springer.com/esm/art%3A10.1038%2Fs41467-025-67136-2/MediaObjects/41467_2025_67136_MOESM3_ESM.csv. Corresponding metatranscriptomic and immunological data are available via Table S7.

## SUPPLEMENTAL MATERIAL

Fig. S1 mbio.03645-25-s0001.pdf Selecting optimal mgSs clusters.

Fig. S2 mbio.03645-25-s0002.pdf Selecting optimal mgCST clusters.

Fig. S3 mbio.03645-25-s0003.pdf VISTA performance and implementation.

Fig. S4 mbio.03645-25-s0004.pdf VISTA mgCSTs and VALENCIA CSTs are largely congruent using taxonomy alone.

Fig. S5 mbio.03645-25-s0005.pdf Community-level transcriptional activity expression.

Fig. S6 mbio.03645-25-s0006.pdf Concentrations of four immune mediators across select VISTA mgCSTs.

Legends mbio.03645-25-s0007.docx Supplemental figure legends.

Supplemental Tables mbio.03645-25-s0008.xlsx Tables S1 to S7.

ASM does not own the copyrights to Supplemental Material that may be linked to, or accessed through, an article. The authors have granted ASM a non-exclusive, world-wide license to publish the Supplemental Material files. Please contact the corresponding author directly for reuse.
