# Supplementary figures and images for: Not all vaginal microbiomes are equal: functional context shapes immune landscapes
Source: mBio. 2026 Feb 5;17(3):e03645-25. doi: 10.1128/mbio.03645-25 (PMC12977605; doi:10.1128/mbio.03645-25)

Avg. silhouette width

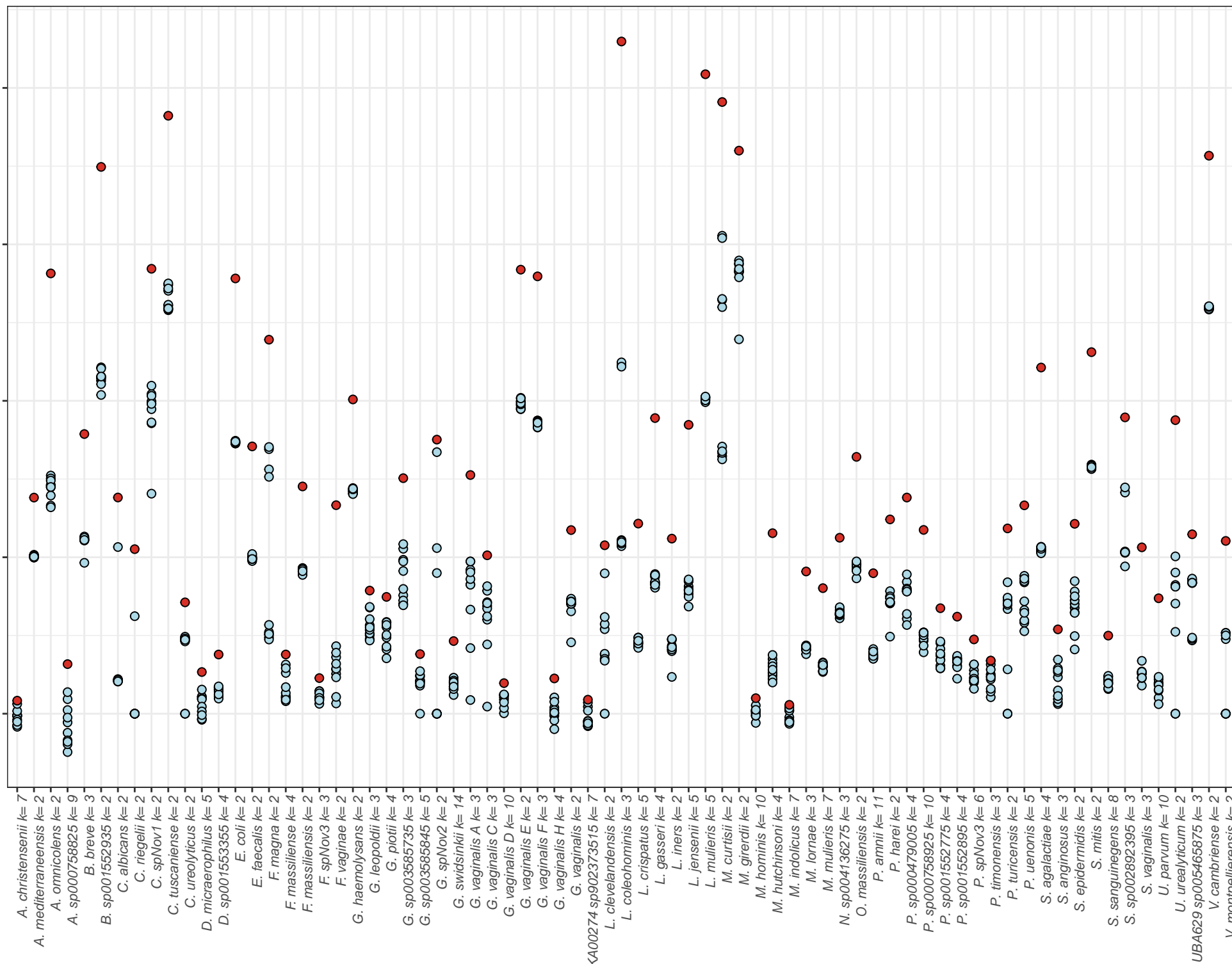

Data type

- Observed
- Randomized

Supplement: Fig. S1 — Selecting optimal mgSs clusters. [file mbio.03645-25-s0001.pdf]

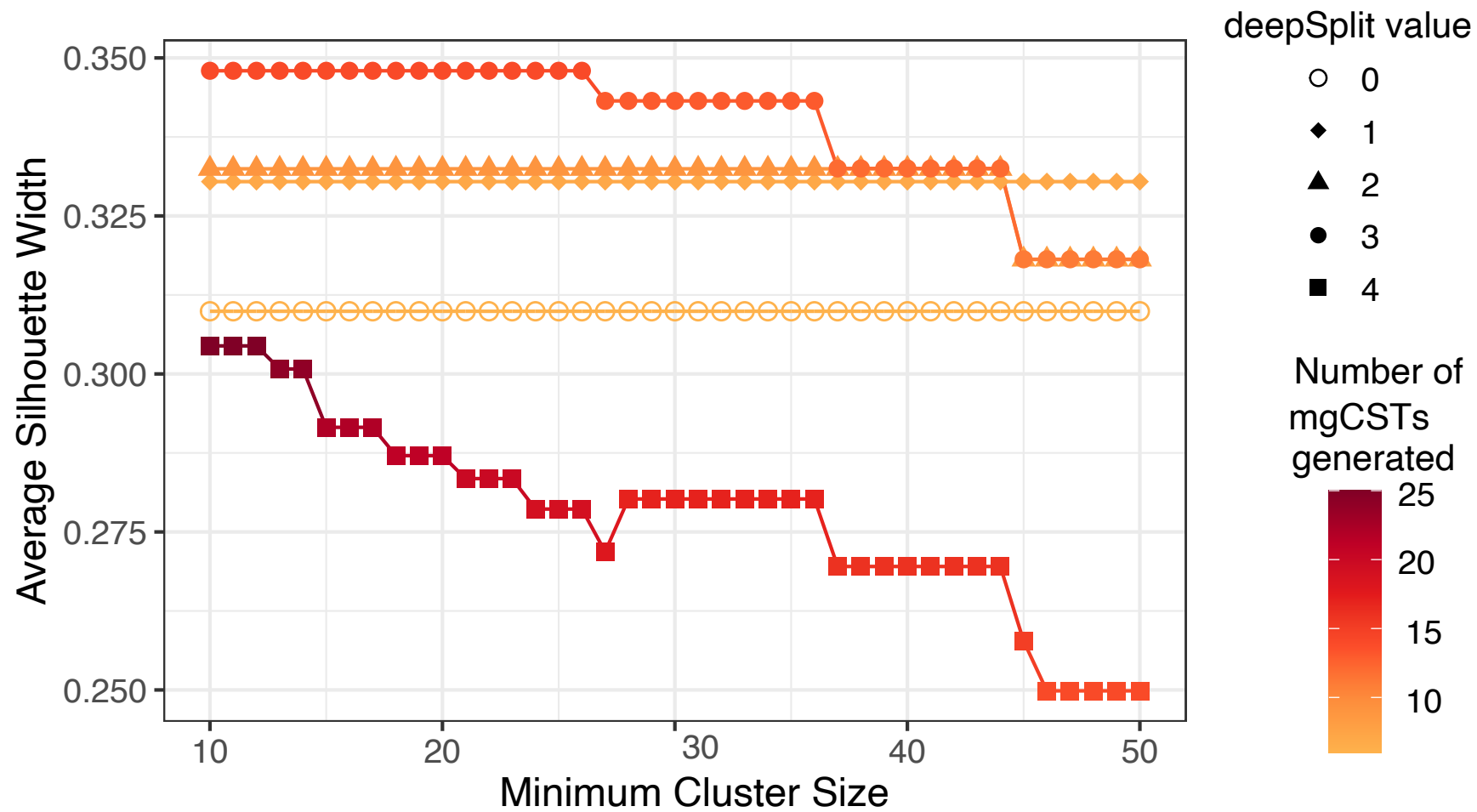

Supplement: Fig. S2 — Selecting optimal mgCST clusters. [file mbio.03645-25-s0002.pdf]

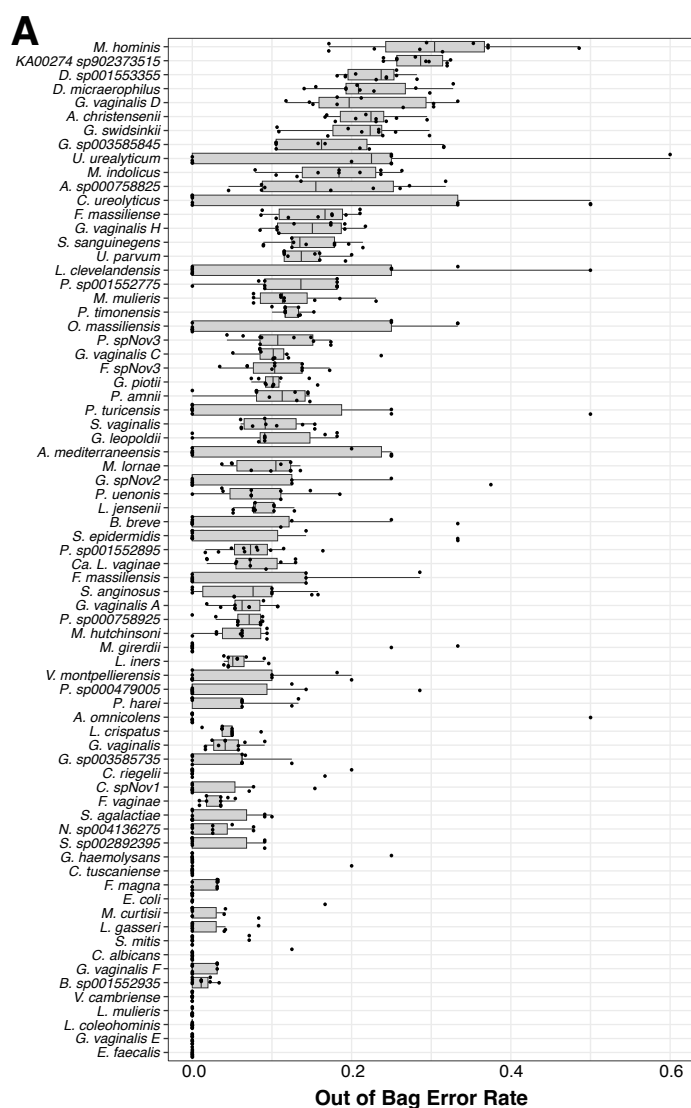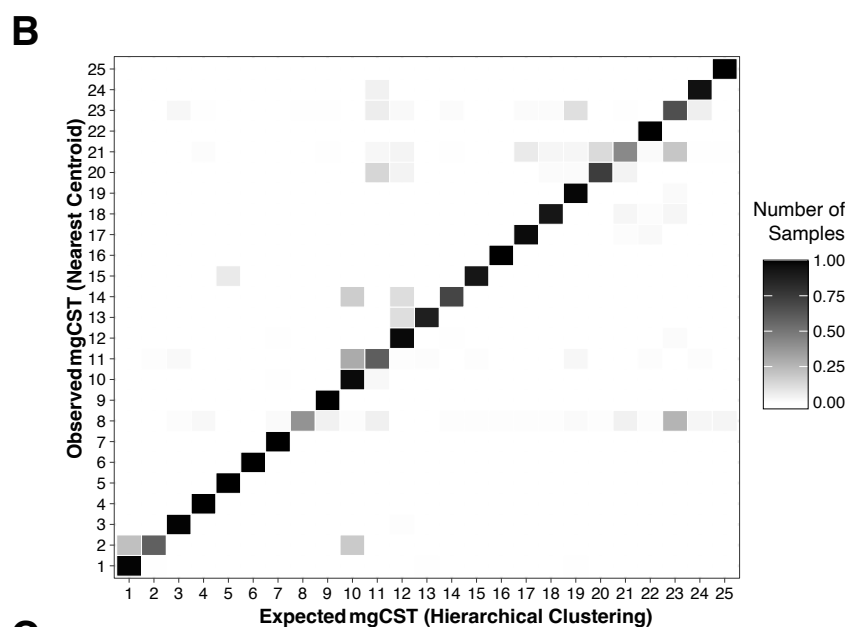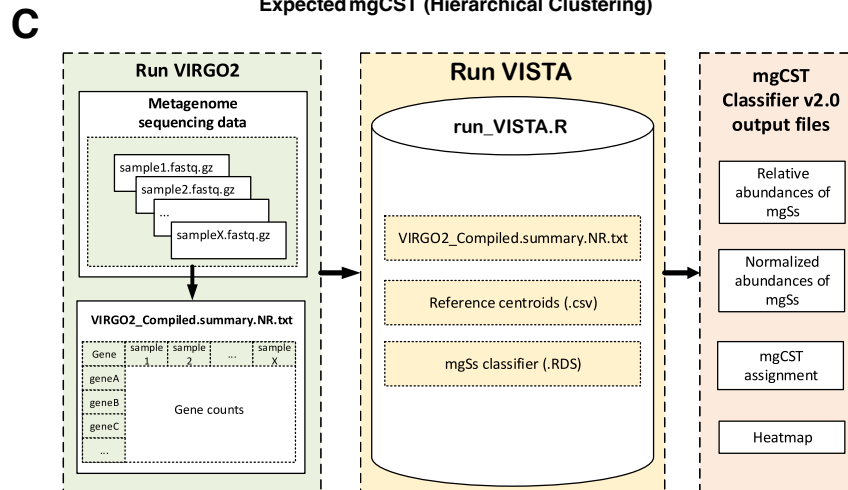

Supplement: Fig. S3 — VISTA performance and implementation. [file mbio.03645-25-s0003.pdf]

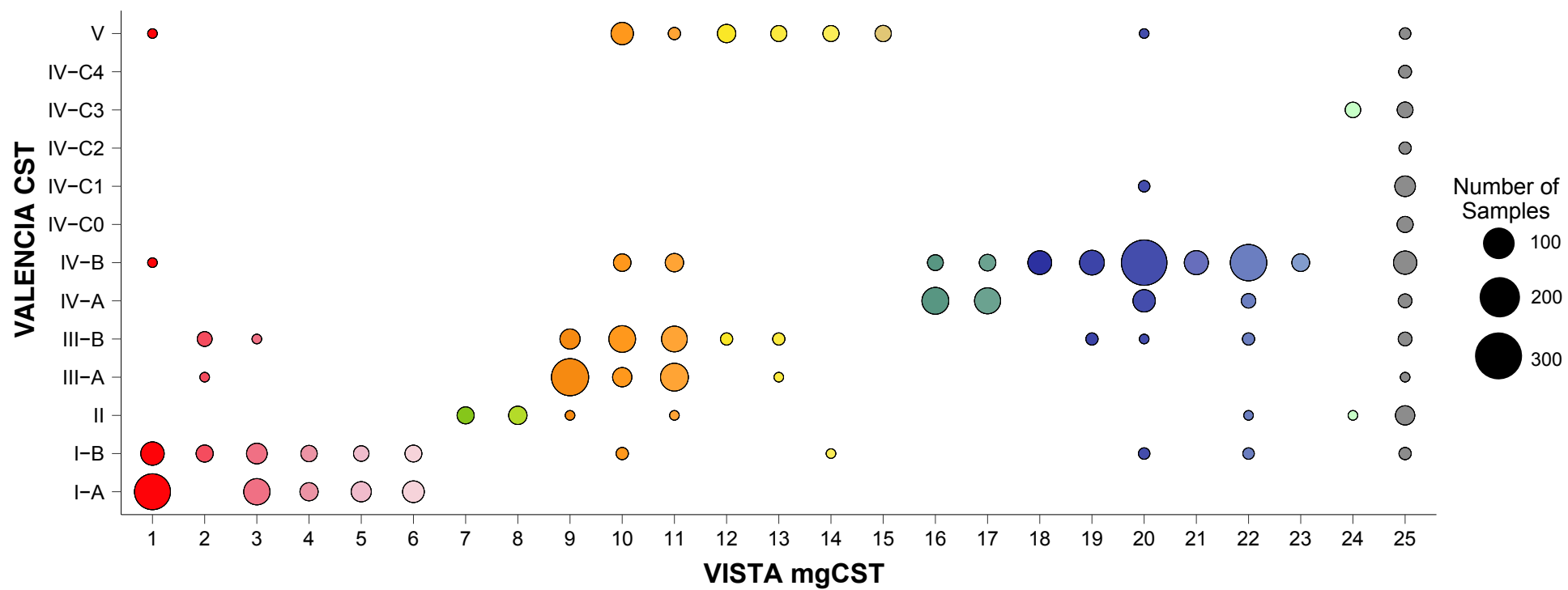

Supplement: Fig. S4 — VISTA mgCSTs and VALENCIA CSTs are largely congruent using taxonomy alone. [file mbio.03645-25-s0004.pdf]

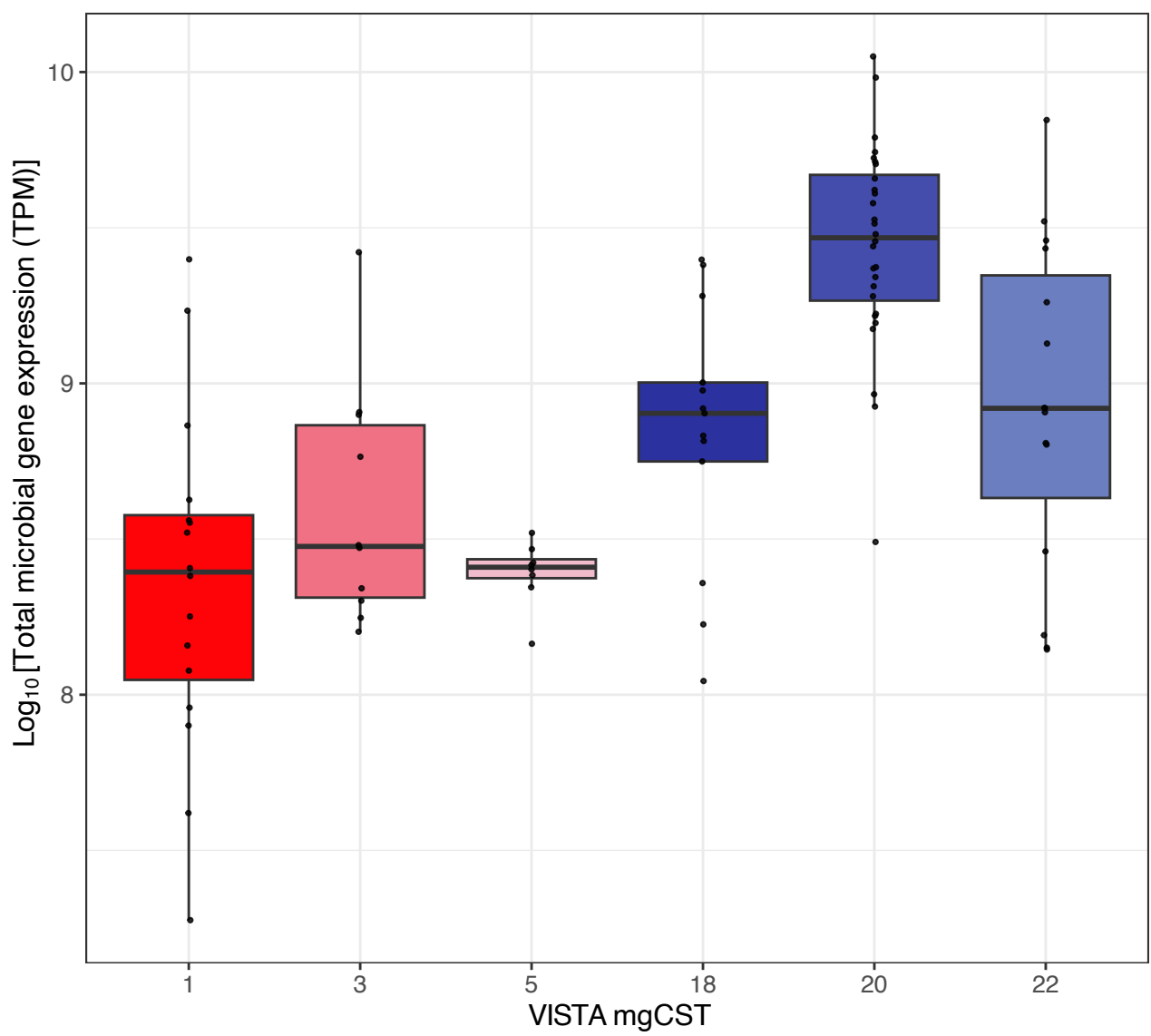

Supplement: Fig. S5 — Community-level transcriptional activity expression. [file mbio.03645-25-s0005.pdf]

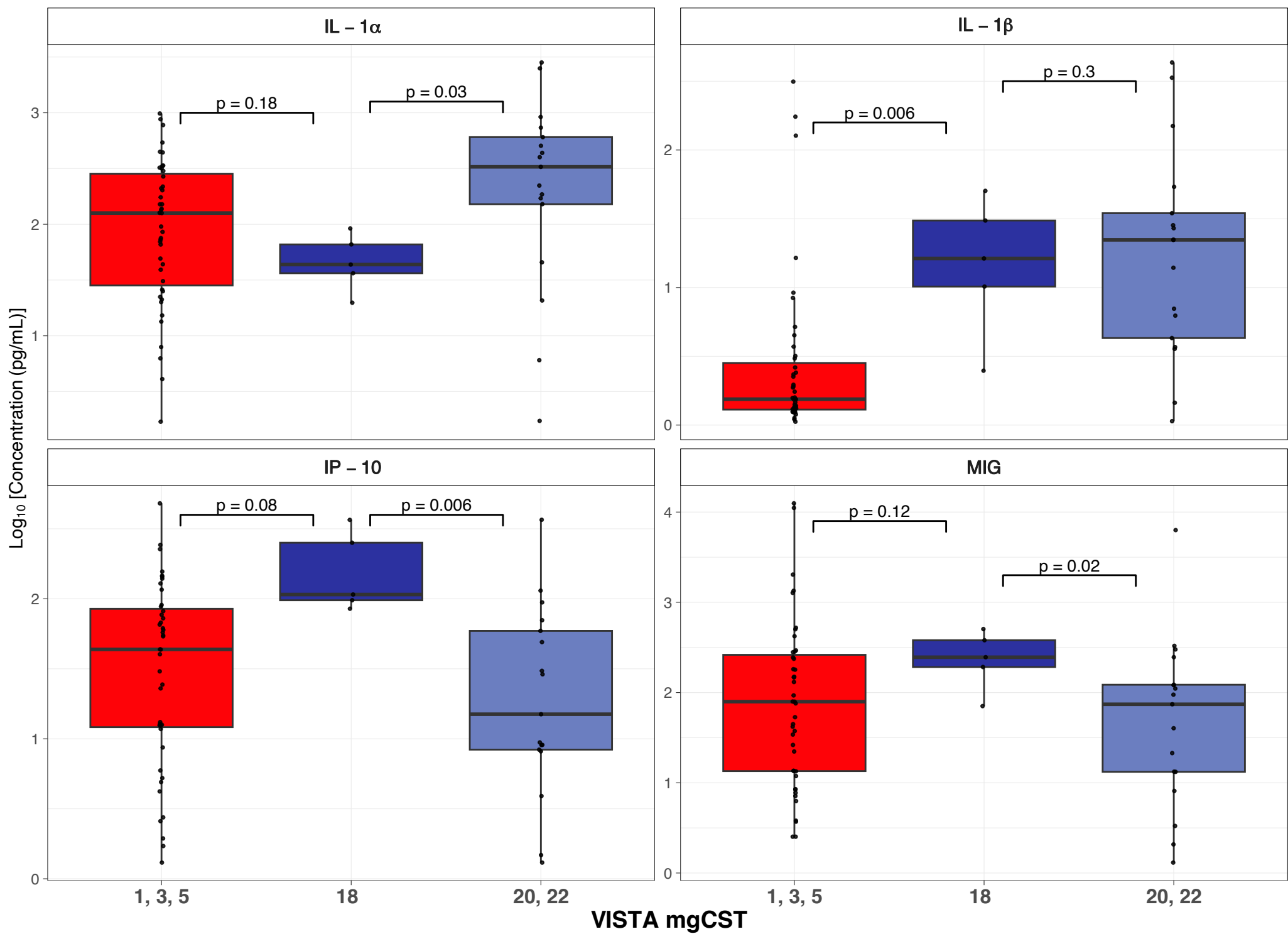

Supplement: Fig. S6 — Concentrations of four immune mediators across select VISTA mgCSTs. [file mbio.03645-25-s0006.pdf]
