# Supplementary material for: Not all vaginal microbiomes are equal: functional context shapes immune landscapes
Source: mBio. 2026 Feb 5;17(3):e03645-25. doi: 10.1128/mbio.03645-25 (PMC12977605; doi:10.1128/mbio.03645-25)
Supplement: Legends — Supplemental figure legends. [file mbio.03645-25-s0007.docx]

**Figure S1.** Comparison of VOG-based mgSs clusters generated from the observed and randomized data for all species.

**Figure S2.** The average silhouette width for each minimum cluster size tested with DynamicTreeCut to define the optimal number of VISTA mgCSTs.

**Figure S3. (A)** Out-of-bag error rates for VOG-based mgSs clusters from each species. **(B)** Confusion plot of observed and expected VISTA mgCSTs. **(C)** Schematic for running VISTA to assign VOG-based mgSs and VISTA mgCSTs.

**Figure S4.** Comparison of VOG-mgCST and VALENCIA-based CST assignments.

**Figure S5.** Log10-transformed total microbial gene expression across VISTA mgCSTs. Expression values represent the sum of TPM-normalized gene expression from all species within each sample and are grouped by mgCST.

**Figure S6.** Log10-transformed concentrations (pg/mL) of immune markers IL-1α, IL-1β, IP-10, and MIG in samples from BioProject PRJNA797778, stratified by VISTA mgCST. ANOVA-derived p-values reflect comparisons between mgCST 18 and L. crispatus-dominated mgCSTs (1, 3, 5), as well as between mgCST 18 and Gardnerella-dominated mgCSTs (20, 22), for each immune marker.
